# Antitumor and anti-metastatic effects of cyclooxygenase-2 inhibition by celecoxib on human colorectal carcinoma xenografts in nude mouse rectum

**DOI:** 10.3892/or.2012.1885

**Published:** 2012-06-26

**Authors:** ITASU NINOMIYA, NOBORU NAGAI, KATSUNOBU OYAMA, HIRONORI HAYASHI, HIDEHIRO TAJIMA, HIROHISA KITAGAWA, SACHIO FUSHIDA, TAKASHI FUJIMURA, TETSUO OHTA

**Affiliations:** Gastroenterologic Surgery, Department of Oncology, Division of Cancer Medicine, Graduate School of Medical Science, Kanazawa University, Ishikawa 920-8641, Japan

**Keywords:** celecoxib, lymph node metastasis, lung metastasis, angiogenesis, prostaglandin E_2_, apoptosis, rectal xenograft model

## Abstract

We examined the effects of the preferential cyclooxygenase-2 (COX-2) inhibitor celecoxib on tumorigenesis, angiogenesis, apoptosis, vascular endothelial growth factor (VEGF) protein expression and metastasis in HT-29 human colorectal carcinoma cell xenografts in nude mouse rectum. COX-2 mRNA expression was examined in the xenograft and metastatic sites. The antitumor effect of celecoxib in the xenografts was evaluated by measuring the weight of the peri-ano-rectal tumor. The anti-metastatic effect of celecoxib was assessed by quantification of lymph node and lung metastases by amplification of a cancer-related human DNA by TaqMan PCR. The effects of celecoxib on angiogenesis, apoptosis, prostaglandin E_2_ (PGE_2_) production and VEGF protein expression in the xenografts were evaluated by means of microvessel density (MVD) counting, terminal deoxynucleotidyl transferase-mediated nick end labeling assay, quantitative enzyme-linked immunosorbent assay and western blotting, respectively. The rectal xenograft model showed lymph node and lung metastases with enhanced expression of COX-2 mRNA in each organ. Celecoxib inhibited rectal xenograft growth in a dose-dependent manner as follows: 150 ppm, 33.0% (p=0.000220); 750 ppm, 46.4% (p=0.0000292); 1500 ppm, 63.4% (p=0.0000109). Celecoxib inhibited lymph node metastasis in a dose-dependent manner as follows: 150 ppm, 86.7% (p=0.0263); 750 ppm, 90.3% (p=0.00638); 1500 ppm, 96.0% (p=0.000894). Celecoxib also inhibited lung metastasis as follows: 750 ppm, 53.3% (p=0.0107); 1500 ppm, 78.3% (p=0.00022). Celecoxib (1500 ppm) significantly inhibited PGE_2_ production by 68.4% (p=0.000157) and MVD counting by 48.2% (p=1.3×10^−12^) and induced apoptosis 2.5-fold (p=3.0×10^−14^) in the rectal xenograft. Celecoxib suppressed VEGF protein expression in the rectal xenograft. These studies demonstrate that celecoxib reduces the growth and metastatic potential of colorectal carcinoma in mice through COX-2 inhibition, anti-angiogenesis and apoptosis induction. The studies using HT-29 human colorectal carcinoma cell xenografts in nude mouse rectum also provide important information that supports that the COX-2 inhibitor celecoxib has a high potential for use as a clinical agent for inhibition of hematological and lymphatic metastases of colorectal cancer.

## Introduction

Colorectal cancer (CRC) is one of the most common internal malignancies and is one of the leading causes of cancer-related morbidity and mortality in the world. Therefore, increasing efforts are being focused on developing more effective screening and prevention measures for CRC. Several epidemiological studies have shown that regular use of aspirin or other non-steroidal anti-inflammatory drugs (NSAIDs) results in 40–50% reduced risk of colorectal cancer (1–3). Furthermore, NSAIDs cause regression of pre-existing adenomas in patients with familial adenomatous polyposis (4) and significantly inhibit tumor growth in animal models of CRC (1,5). The mechanism by which aspirin and NSAIDs inhibit cyclooxygenase (COX) activity was first described by Vane (6). This observation led to the hypothesis that both the toxicity and efficacy of NSAIDs are mediated through the inhibition of COX-mediated prostaglandin (PG) synthesis (7). In the early 1990s, several groups reported the discovery of two COX isoforms, COX-1 and COX-2. COX-1 is constitutively expressed in normal intestine and appears to function as a physiological regulatory enzyme in most tissues, including the gastric mucosa, the kidney and platelets. However, the COX-1 level does not change in intestinal tumors. COX-2 is nearly undetectable in most tissues under normal physiological conditions (7) and is a strongly inducible form that is involved in growth proliferation and the inflammatory response (8,9). COX-2 protein expression is enhanced in human CRC (10). COX-2-derived PG was found to play a key role in colorectal tumorigenesis in ApcΔ16 knockout mice (11). The invasiveness of colon cancer cells was increased by COX-2 transfection in association with PG production and was reversed by sulindac sulfide, a COX inhibitor (12). COX-2 and COX-2-derived PGs mediated tumor growth and metastasis in animal models by inducing the formation of blood vessels (13). Specific COX-2 inhibition, either by pharmacological intervention or by targeted knockout of the COX-2 gene, has been shown to effectively reduce the growth of xenograft tumor in mice (3,13).

Celecoxib is a drug that was designed to treat the signs and symptoms of adult arthritis (14). Celecoxib inhibits the inflammatory COX-2 enzyme at therapeutic doses in humans that do not lower gastrointestinal prostaglandin levels associated with mucosal protection (15). Celecoxib blocked angiogenesis and inhibited lung and colon cancer xenograft growth in mice (13). Several recent reports have suggested that COX-2 expression plays an important role in haematogenous metastasis of CRC to the liver (16,17). These previous reports demonstrated that celecoxib has anti-metastatic effects when cancer cells were pretreated with celecoxib. We previously developed an animal model of orthotopic transplanted CRC associated with spontaneous lymph node and hematological metastases by intra-rectal injection of tumor cells in nude mouse rectum, consistent with the progression of CRC in humans (18).

The aim of the present study was to investigate whether oral administration of COX-2 inhibitor celecoxib prevents tumorigenesis and spontaneous lymph node and lung metastases of CRC by using a rectal xenograft model identical to human CRC.

## Materials and methods

### Animals

All animal experiments were performed according to Kanazawa University’s standard guidelines. Nude mice (BALB/cAnNCrj-nu/nu) aged 5 weeks were purchased from Charles River Japan, Inc. (Kanagawa, Japan). After at least 1 week of observation, the mice were used for this study at the age of 6–7 weeks. All mice were housed in the Laboratory for Animal Experiments, Research Institute, Kanazawa University School of Medicine, under laminar airflow conditions. Housing was temperature-controlled, with a 12-h/12-h light/dark cycle.

### Cell line

The human colon cancer cell line HT-29 was used. Cells were cultured in RPMI-1640 medium (RPMI) (Nissui, Tokyo, Japan) supplemented with 10% heat-inactivated fetal bovine serum (JRH Biosciences, Lenexa, KS) and antibiotics (100 U/ml penicillin and 100 μg/ml streptomycin), at 37°C in a humidified atmosphere of 95% air/5%CO_2_. The cells were passaged when confluent.

### Human cancer xenograft models

Log-phase HT-29 human colon carcinoma cells were harvested with Trypsin EDTA, washed three times with RPMI and resuspended at a cell density of 1×10^7^/ml in RPMI. Animals were anaesthetized with ether and placed in a supine position. To prevent colonic obstruction resulting from rectal tumor progression, a 7-mm cut of the anterior end of the ano-rectal wall in the ano-rectal region was performed. Tumor cells (1×10^6^/0.1 ml/mouse) in RPMI were slowly injected submucosally into the posterior wall with a 27-gauge needle.

### Preparation of celecoxib (SC-58635)

The preferential COX-2 inhibitor, celecoxib, was purchased from Pfizer Japan Inc. (Tokyo, Japan). We prepared chows containing 150, 750 or 1500 ppm celecoxib. The mice received celecoxib chow for 4 weeks starting at 2 weeks after tumor cells inoculation. Control mice received normal chow.

### Antitumor efficacy

To evaluate the antitumor and anti-metastatic effects of celecoxib, the mice were sacrificed 6 weeks after tumor cells inoculation. The antitumor effect of celecoxib in the xenograft was evaluated by measuring the weight of the peri-ano-rectal tumor. To evaluate metastasis, the lymph nodes around the abdominal aorta and lung were excised. DNA from each specimen was extracted by the standard proteinase K digestion-phenol chloroform extraction method as described previously (19). The human β-globin-related sequence in the extracted DNA was amplified by quantitative PCR by using the 5′ nuclease assay and an ABI PRISM 7700 Sequence Detector (TaqMan) (PE Biosystems Japan, Tokyo, Japan). One microgram of each DNA sample was amplified in a 50-μl reaction mixture consisting of 200 nM of forward and reverse primers, 100 nM of probe and TaqMan Universal Master Mix (PE Biosystems Japan), which contained Ampli-Taq Gold DNA polymerase, reaction buffer, dNTP, dUTP and AmpErase uracil-N-glycosylase. The PCR reaction was carried out on an ABI 7700 with the following thermo-cycler conditions: 50°C for 2 min, 95°C for 5 min, followed by 45 cycles of 95°C for 15 sec and 60°C for 1 min. Data were analyzed by using Sequence Detection Software (PE Biosystems Japan). The number of metastasized tumor cells in the excised whole organ was calculated from the standard curve of a serial dilution series of HT-29 DNA after quantitative PCR as described previously (18). Primer and probe sequences for human β-globin gene amplification were as follows: forward, CACT GACTCTCTCTGCTATTGGTC; reverse, AGGAGTGGAC AGATCCCCAAA; TaqMan probe, 6FAM5′-CTACCCTTG GACCCAGAGGTTCTTTGAGTC-3′ TAMRA.

### Quantification of human COX-2 mRNA expression

We evaluated the human COX-2 mRNA level in the rectal xenograft. The primary xenograft, para-aortic lymph nodes and lung tissue were obtained from 5 mice bearing rectal tumors 6 weeks after tumor cell inoculation. RNA was extracted by using Isogen systems (Nippon Gene Co., Ltd., Tokyo, Japan). After heat denaturation at 68°C for 15 min with 500 pmol of the oligo(dT) primer, 10 μg of RNA was reverse-transcribed at 42°C for 60 min into first-strand cDNA in reverse-transcription solution [400 units of Moloney murine leukemia virus reverse transcriptase (Invitrogen Japan K.K., Tokyo, Japan), 50 mM Tris-HCl (pH 8.3), 75 mM KCl, 3 mM MgCl_2_, 0.01 M DTT, 0.5 mM of each dNTP and 16 units of RNasin (Promega, Madison, WI)] with a total volume of 100 μl. A reverse-transcribed cDNA solution corresponding to 100 ng of total RNA was amplified quantitatively with primers and a probe specific for the targeted cDNA with an ABI PRISM 7700 Sequence Detector (TaqMan). The final concentrations of reaction components, except for template cDNA, and thermo-cycler conditions were the same as those for the human β-globin-related sequence amplification described above. Internal standard gene expression was examined by using TaqMan GAPDH control reagents and TaqMan rodent GAPDH control reagents VIC (PE Biosystems Japan). After standardization by internal standard gene expression, human COX-2 gene expression was calculated relative to the *in vitro* COX-2 expression level of HT-29 cells. The primer and probe sequences for COX-2 mRNA amplification were as follows: forward, GGT TGCTGGTGGTAGGAATGTT; reverse, CATAAAGCGTT TGCGGTACTCA; TaqMan probe, 6FAM5′-CCGCAGTAC AGAAAGTATCACAGGCTTCCA-3′ TAMRA.

### PGE_2_ measurement

The concentration of PGE_2_ in the rectal xenograft was determined by quantitative enzyme-linked immunosorbent assay (ELISA) (Cayman Chemical, Ann Arbor, MI), according to the manufacturer’s instructions.

### Quantification of microvessel density (MVD)

The MVD in the rectal xenograft was determined using immunohistochemistry with an anti-mouse CD31 antibody. The tumor xenografts were sliced to 5-μm sections at the maximum diameter. The sliced specimens were fixed in acetone at 4°C overnight. After rinsing with PBS, they were immersed in 30% sucrose-PBS overnight for at least 4 h until they sunk. They were embedded in Cryomold (Sakura Finetek Japan, Tokyo, Japan) by Tissue-Tek O.C.T. compound (Sakura Finetek Japan). They were frozen on dry-ice acetone. They were sectioned with a cryothome at a thickness of 7 μm and mounted on precoated slides. These slides were incubated at 4°C overnight with rat anti-mouse CD31 antibody (BD Biosciences Pharmingen, San Diego, CA). Then, the slides were incubated in goat anti-rat IgG HRP (Santa Cruz Biotechnology, Santa Cruz, CA) as the secondary antibody. Vascularity was measured by the average number of vessels per field counted in 8 random areas at ×20 magnification. Only areas of viable tumor tissues were imaged; necrotic regions and overlying subdermal regions were excluded. MVD was calculated as the average number of vessels per mm^2^.

### Western blot analysis

We examined the effect of celecoxib treatment on the expression of mouse rectal xenograft proteins involved in angiogenesis. Rectal xenograft samples from 1500 ppm celecoxib-treated and control groups were assessed for the expression of vascular endothelial growth factor (VEGF) protein by specific immunoblot analyses. The protein concentration in each sample was measured using a Bio-Rad protein assay kit II (Bio-Rad Laboratory, Richmond, CA). For SDS-PAGE, 10 μg of protein from each sample was loaded on 12% polyacrylamide gels. Proteins were transferred to a polyvinylidene difluoride membrane with a tank transfer system (Bio-Rad Laboratory), and then blocked with a buffer containing 5% low fat skim milk and 0.1% Tween-20 in tris-buffered saline (TBST) at room temperature for 1 h. We used rabbit anti-human VEGF which cross-reacted with VEGF of mouse as the primary antibody (Santa Cruz Biotechnology). The primary antibody was diluted in TBST containing 5% skim milk at a dilution of 1:2000. The membrane was incubated with the primary antibody overnight at 4°C. After washing three times with TBST, the membrane was incubated with a horseradish peroxidase-conjugated secondary antibody (0.02 μg/ml in TBST) at room temperature for 1 h. We used goat anti-rabbit IgG HRP (1:5000 dilution; Chemicon International, Temecula, CA) as the secondary antibody. Detection of chemiluminescence was performed with ECL western blot detection kits (Amersham, Little Chalfont, UK), according to the manufacturer’s instructions.

### Measurement of apoptosis

We examined the effect of celecoxib treatment on the degree of apoptosis of mouse rectal xenograft tumor cells by terminal deoxynucleotidyl transferase-mediated nick end labeling (TUNEL) assay. Rectal xenografts were embedded in paraffin and sliced to 5-μm sections. The sections were dewaxed and rehydrated using routine procedures. Apoptosis *in situ* detection kit was purchased from Wako (Osaka, Japan). Apoptosis was assessed by the average number of apoptotic cells per field counted in 8 random areas at ×40 magnification. The average number of tumor cells per field was also determined at ×40 magnification. The apoptotic index was calculated as follows: apoptotic index (%) = (no. of apoptotic cells/no. of total tumor cells × 100).

### Statistical analysis

The effect of celecoxib was assessed for associations with number of metastasized tumor cells, PGE_2_ production, VEGF production, MVD counting and apoptosis index by using the Mann-Whitney’s U test. P<0.05 was considered to be statistically significant.

## Results

### Rectal xenograft model

**Figure 1:**
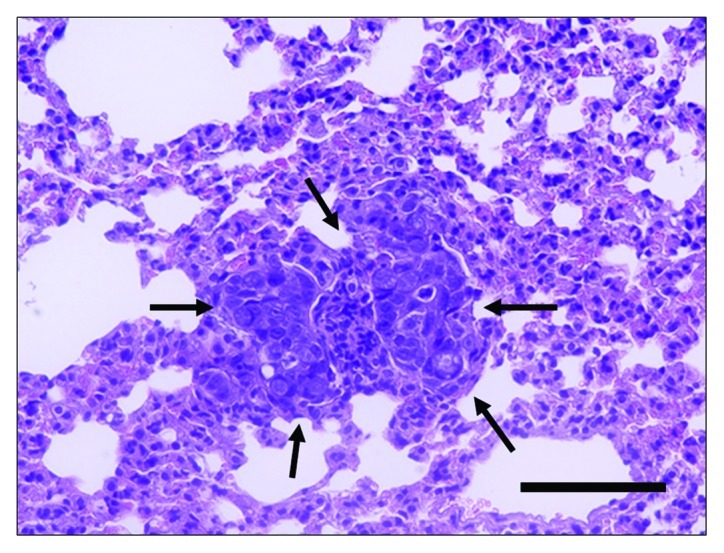
Microscopic findings of the lung of a mouse with the HT-29 cell rectal xenograft. Black arrows show lung metastasis. Scale bar, 100 μm.

Macroscopically, a rectal xenograft developed into a rectal tumor within 1 week after tumor cell inoculation. The HT-29 xenograft produced a locally aggressive rectal tumor and subsequently showed lymph node metastasis around the abdominal aorta 6 weeks after tumor cell inoculation. No apparent metastatic nodules were observed macroscopically in the lung, liver, spleen, kidneys, or peritoneum. Histologically, multiple tiny metastatic foci in the lungs were observed 6 weeks after tumor cell inoculation (Fig. 1).

### Quantification of human COX-2 mRNA expression

Expression levels of human COX-2 mRNA from tumor cells in the primary xenograft tissue, lymph nodes and lungs from mice bearing rectal xenografts were analyzed (Table I). COX-2 mRNA expression was increased 3.63-, 3.32- and 37.6-fold in the rectal xenograft tissue, lymph nodes, and lungs to that in the *in vitro* HT-29 cells, respectively.

### Antitumor efficacy of celecoxib in xenografts

**Figure 2:**
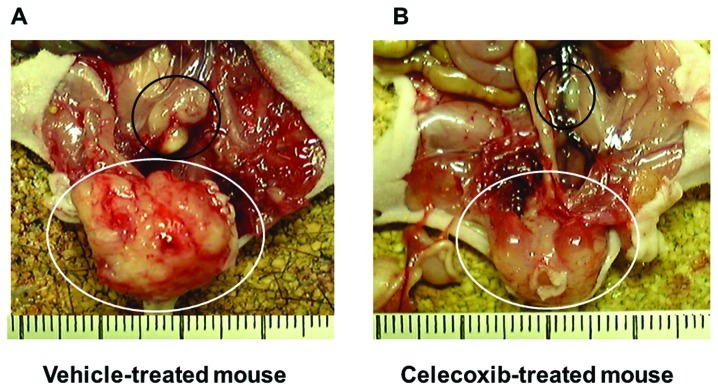
Macroscopic findings of a rectal xenograft model 6 weeks after tumor inoculation in vehicle-treated mouse (A) and celecoxib-treated mouse (B). White ring shows peri-ano-rectal tumor and black ring shows para-aortic lymph node metastasis.

**Figure 3:**
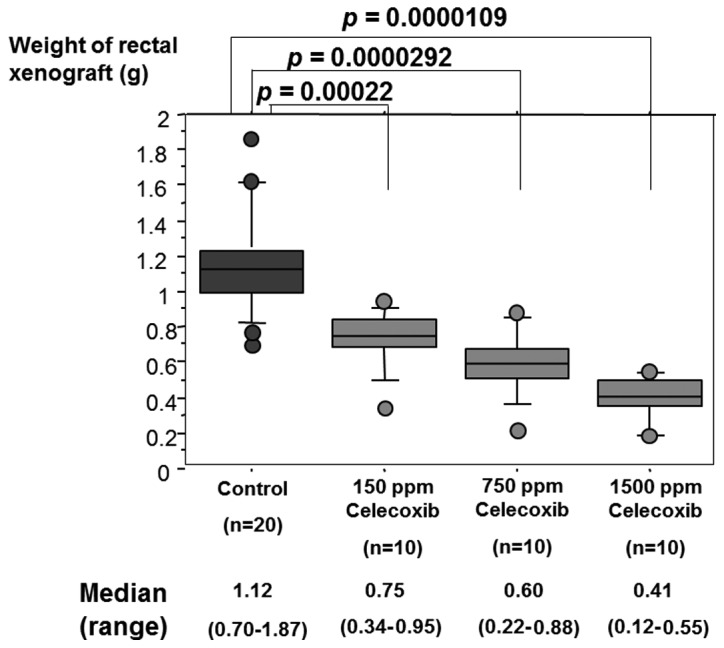
Effect of celecoxib on HT-29 rectal xenograft growth. The weight of the rectal xenograft of celecoxib-treated mice and vehicle-treated control mice bearing the HT-29 rectal xenograft was measured. Celecoxib repressed growth of rectal xenograft in a dose-dependent manner.

Celecoxib macroscopically inhibited xenograft growth and abdominal lymph node metastasis (Fig. 2). The antitumor activity of celecoxib in rectal xenograft was examined by comparing the wet weight of each xenograft. Celecoxib suppressed growth of rectal xenograft in a dose-dependent manner. Celecoxib (150, 750 and 1500 ppm) significantly inhibited HT-29 xenograft growth in comparison with normal chows by 33.0% (p=0.00022), 46.4% (p=0.0000292) and 63.4% (p=0.0000109), respectively (Table II, Fig. 3).

### Anti-metastatic efficacy of celecoxib

**Figure 4:**
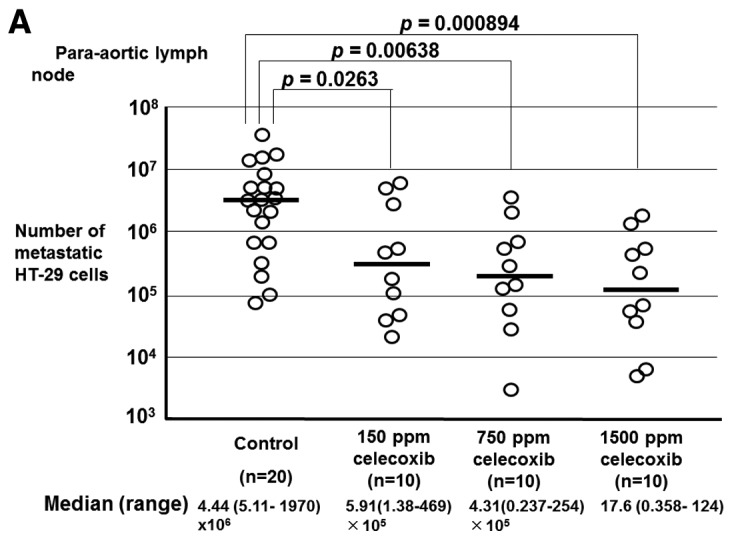

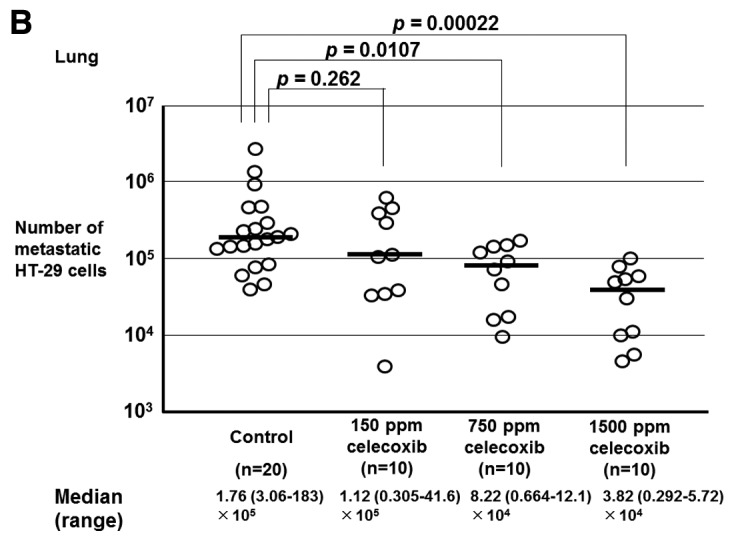
Quantification of metastasized tumor cell amounts in para-aortic lymph node (A) and lung (B) of mice with the HT-29 rectal xenografts treated with various concentrations of celecoxib. The amount of metastasized tumor cells was calculated by amplification of the human β-globin gene and shown as the number of tumor cells in the organ. Celecoxib inhibited lymph node and lung metastases in mice with rectal xenograft in a dose-dependent manner.

The anti-metastatic activity of celecoxib was examined by amplifying the human β-globin-related sequence in the lymph nodes and the lung of rectal xenograft mice. Quantification of cancer metastasis by calculating metastasized tumor cells from the quantitatively amplified β-globin gene revealed that 150, 750 or 1500 ppm celecoxib significantly inhibited lymph node metastasis and 750 or 1500 ppm celecoxib significantly inhibited lung metastasis of HT-29 cells in rectal xenograft (Fig. 4). Assessment of metastasis by the median level of quantified metastatic cells revealed that the inhibition ratios of lymph node metastasis by 150, 750 and 1500 ppm celecoxib were 86.7% (p=0.0263), 90.3% (p=0.00638) and 96.0% (p=0.000894), respectively. Inhibition ratios of lung metastasis by 750 and 1500 ppm celecoxib were 53.3% (p=0.00107) and 78.3% (p=0.00022), respectively (Table II). The results also demonstrated that celecoxib suppressed lymph node and lung metastases in a dose-dependent manner.

### Anti-angiogenesis efficacy of celecoxib

**Figure 5:**
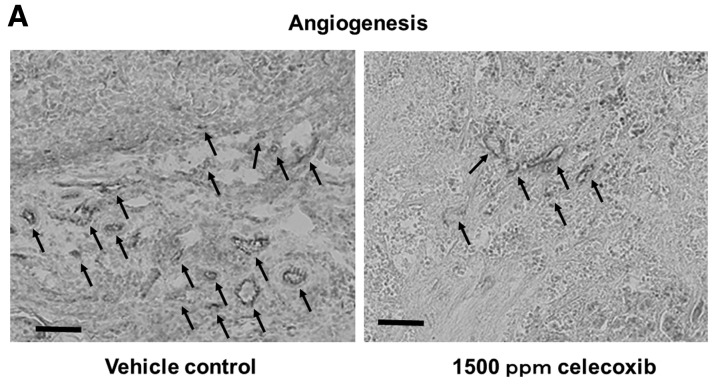

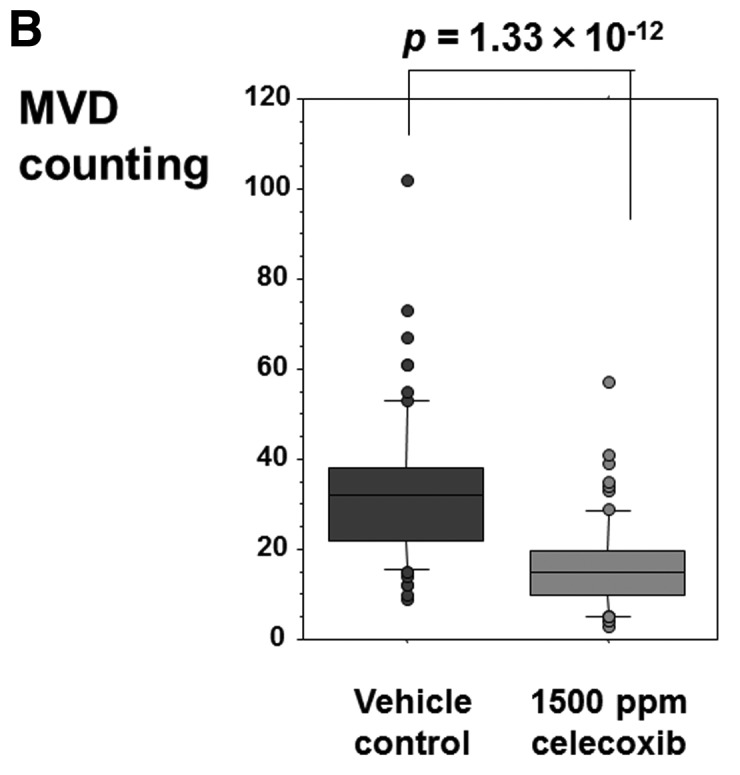
Effect of celecoxib on angiogenesis in rectal xenograft. (A) Representative micrograph of immunohistochemistry with an anti-mouse CD31 antibody of the rectal xenograft of a mouse treated with vehicle and celecoxib-treated mouse. Black arrows show CD31-positive microvessels. Scale bar, 100 μm. (B) Vascularity was measured by the average number of vessels per field counted in 8 random areas at ×20 magnification. MVD was calculated as the average number of vessels per mm^2^. Celecoxib significantly inhibited angiogenesis in the rectal xenograft.

The anti-angiogenetic activity of celecoxib was examined by MVD count in rectal xenografts. Celecoxib (1500 ppm) significantly reduced MVD (Fig. 5). Assessment of angiogenesis by comparison of the median level of MVD in normal chow-fed mice with 1500 ppm celecoxib chows-treated mice revealed that celecoxib inhibited angiogenesis in the rectal xenograft by 48.2% (p=1.3×10^−12^) (Table III).

### Efficacy of celecoxib treatment on PGE_2_ production

**Figure 6:**
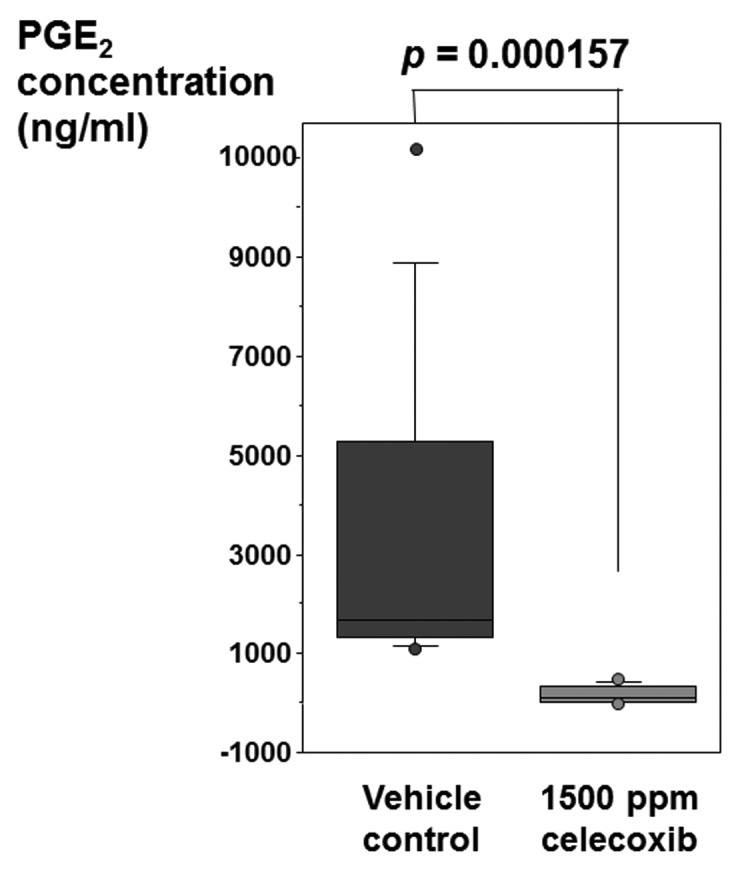
Effect of celecoxib on PGE_2_ production in rectal xenograft as measured by quantitative ELISA. Celecoxib significantly suppressed PGE_2_ production in the rectal xenograft.

Rectal xenograft samples from celecoxib-treated and control mice were assessed for PGE_2_ concentration by quantitative ELISA. The concentration of PGE_2_ was reduced in the rectal xenograft of mice treated with celecoxib in comparison with control animals (Fig. 6). Celecoxib (1500 ppm) inhibited PGE_2_ production in the rectal xenograft by 68.4% (Table III). Celecoxib significantly suppressed PGE_2_ production in the xenograft (p=0.000157).

### Efficacy of celecoxib treatment on VEGF expression

**Figure 7:**
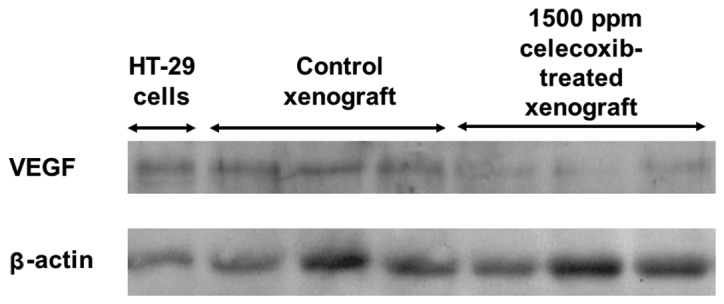
Effect of celecoxib on VEGF expression in rectal xenograft as detected by western blotting. HT-29 human colon cancer cells and rectal xenograft of vehicle-treated control mice showed stronger VEGF expression than rectal xenograft of mice treated with 1500 ppm celecoxib.

We examined the effect of celecoxib treatment on the expression of VEGF which is involved in angiogenesis in rectal xenograft by western blotting. HT-29 human colon cancer cells and control xenograft showed a stronger signal of VEGF protein than 1500 ppm celecoxib-treated xenograft (Fig. 7).

### Efficacy of celecoxib treatment on apoptosis

**Figure 8:**
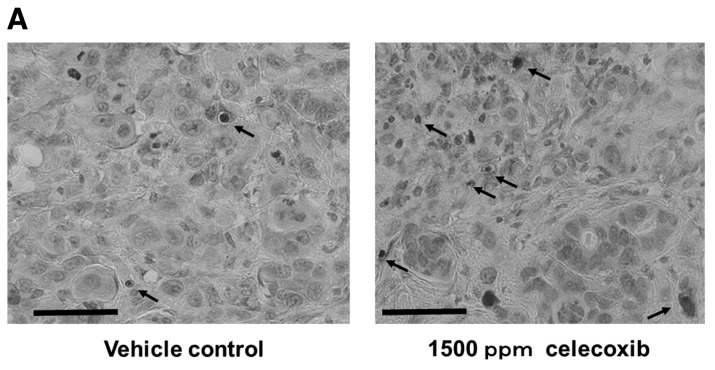

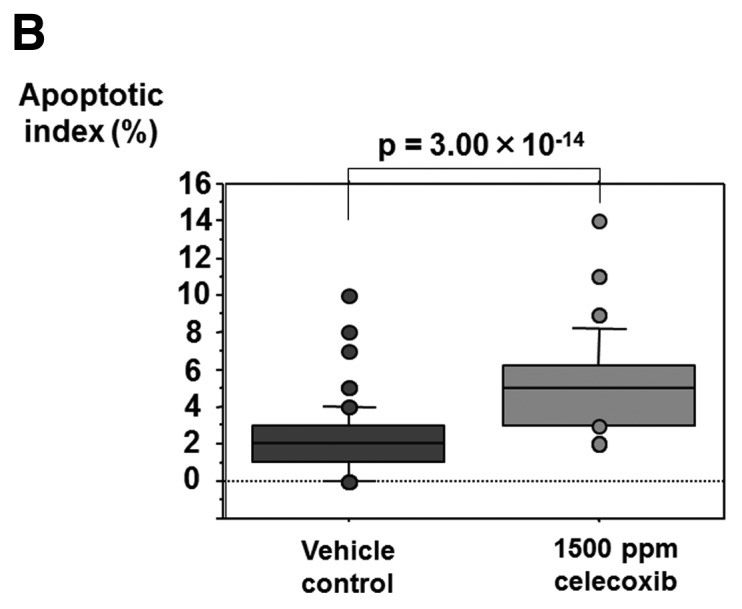
Effect of celecoxib on tumor cell apoptosis in rectal xenograft as detected by TUNEL assay. (A) Representative micrograph of TUNEL assay in the rectal xenograft of a mouse treated with vehicle and celecoxib-treated mouse. Black arrow shows the apoptotic cells. Scale bar, 50 μm. (B) The apoptotic index was calculated as follows: apoptotic index (%) = (no. of apoptotic cells/no. of total tumor cells × 100). Celecoxib significantly induced tumor cell apoptosis.

The TUNEL assay revealed that celecoxib significantly induced apoptosis of HT-29 cells in rectal xenograft (p=3.0×10^−14^) (Fig. 8). Celecoxib induced tumor cell apoptosis consisting of a 2.5-fold increase in tumor cell apoptotic index from 1.64% (0.820–8.20) in vehicle to 4.1% (1.64–11.5) with 1500 ppm celecoxib treatment (Table III).

## Discussion

In the present study, the selective COX-2 inhibitor celecoxib inhibited tumor growth and prevented spontaneous lymph node and lung metastases of CRC in a dose-dependent manner in a mouse rectal xenograft model. Celecoxib inhibited angiogenesis, VEGF expression and PGE_2_ production, and induced tumor cell apoptosis in the rectal xenograft.

In this study, we examined the antitumor efficacy of celecoxib in rectal xenograft tumor. Celecoxib suppressed growth of rectal xenograft in a dose-dependent manner. Because the location of the xenograft in this model is intrinsically near the incident position of rectal carcinoma, we thought that this would be a good model to assess the efficacy of antitumor agents such as celecoxib. Previous reports suggested that COX-2 expression plays an important role in hematogenous metastasis of colorectal carcinomas to the liver (16). Oral administration of COX-2 inhibitors, rofecoxib and JTE-522, reduced the metastatic potential of colon cancer cells injected in the spleen (3,20). Celecoxib suppressed tumor growth and lung metastasis of a rodent mammary cancer and human breast cancer xenograft (21–23). Pretreatment with celecoxib inhibited liver metastasis of colon cancer cells including HT-29 that express a high level of COX-2 in the enforced metastasis model by cancer cell splenic injection (17). These previous reports proved the anti-metastatic effect of celecoxib by pre-treatment of cancer cells with celecoxib or by the non-physiological enforced metastasis model. We previously showed the usefulness of orthotopic transplanted mouse rectal xenograft model of CRC presenting spontaneous lymph node and lung metastases, identical to human rectal cancer (18). This mouse model presented microscopic metastatic foci in the lungs. Therefore, a special technique is needed for detection and quantification of metastatic tumor cells. To detect and quantify the small amount of the metastatic cancer cells, we amplified human β-globin related sequence by TaqMan PCR. Endo *et al*(24) first reported the usefulness of amplifying a human-specific DNA fragment by PCR to detect metastatic human cancer cells in chick embryo. DNA PCR can assess all of the tumor-related signals in the whole tissue, regardless of the size, density and number of tumor colonies. Quantification by DNA amplification is more reliable than reverse transcription-PCR assessment of enzymes originating from the human cancer cells, because the expression levels of enzymes may vary under various tumor conditions. This animal model is useful to assess the anti-metastatic efficacy of novel anti-cancer agents (18). The human colon cancer cell line HT-29 expressed COX-2 enzyme *in vitro*. However, in the model with subcutaneously injected cancer cell xenograft including HT-29 cells xenograft, COX-2 expression was found only in the stromal component without cancer cells, unlike human cancer (25,26). The present study showed that the transplanted xenograft and the metastatic cancer cells in the lymph nodes and lung presented enhanced COX-2 mRNA like human CRC (Table I). Therefore, the mouse rectal xenograft is an optimal model to assess the effect of anti-COX-2 agents. Our present study demonstrated that celecoxib inhibited lymph node and lung metastases of rectal xenograft in a dose-dependent manner.

In this study, we also showed that celecoxib reduced PGE_2_ production and VFGF expression, had an anti-angiogenetic effect, and induced apoptosis in CRC rectal xenograft tumor. These results are consistent with previous reports using various cancer xenografts. Inhibition of COX-2 by celecoxib resulted in loss of intra-tumor PGE_2_ levels and reduced tumor growth with increased apoptosis of both tumor and stromal cells (inflammatory and neovascular) in head and neck xenograft tumors (27). Celecoxib inhibited VEGF expression and reduced angiogenesis and metastasis of human pancreatic cancer that was orthotopically transplanted in nude mice (28). Celecoxib inhibited angiogenesis and VEGF-A expression, and induced the mitochondrial pathway of apoptosis in a murine mammary cancer model (21). Recent studies have confirmed the hypothesis that tumor growth is dependent on angiogenesis. Any significant increase in tumor mass must be preceded by an increase in the vascular supply to deliver nutrients and oxygen to the tumor. COX-2 inhibition leads to reduced conversion of arachidonic acids to PGs, and inhibition of PGE_2_ synthesis is thought to be one of the antitumor mechanisms (29). PGE_1_ and PGE_2_ have the ability to induce angiogenesis in the rat cornea (30). COX-2 and COX-2-derived prostaglandins may play a major role in the development of cancer through numerous biochemical mechanisms, including stimulation of tumor cell growth and neovascularization (13). The antitumor activity of celecoxib may be attributable, at least in part, to a direct effect on host stromal elements, such as the angiogenic vasculature (26). We also showed inhibition of lymph node metastasis in the present study. Although we did not evaluate the effect of celecoxib on lymphangiogenesis, celecoxib might block lymphangiogenesis via downregulation of VEGF-C as reported in lung adenocarcinoma xenograft (31).

Experiments on the kinetics of metastasis using rectal xenograft showed that the initial lymph node and lung metastasis occurred 2 and 4 weeks after tumor cell inoculation, respectively (18). Celecoxib inhibited angiogenesis in the primary tumor. Inhibition of tumor cell dissociation from the primary tumor as well as reduced establishment of metastases by circulating tumor cells might be attributable to the anti-metastatic effect of celecoxib in rectal xenograft model (32).

The ability of celecoxib to block angiogenesis and suppress tumor growth and metastasis suggests a novel application of this anti-inflammatory drug in the treatment of human cancer. The tumor-suppressive action of celecoxib was not associated with noticeable side effects on late wound healing and on the gastrointestinal tract (33). Celecoxib prevents morphine-induced stimulation of COX-2, PGE_2_, angiogenesis, tumor growth, metastasis and mortality without compromising analgesia (34). Therefore, prophylactic use of the drug can be advocated in many clinical situations of cancer treatment, such as residual tumors or contamination of surgical fields by tumor cells. The combination of celecoxib and morphine might also be optimal for analgesia in cancer patients with chronic and severe pain. To prove the usefulness of celecoxib in cancer treatment, clinical trials for various situations in cancer treatment are warranted.

## Figures and Tables

**Table I:** Quantification of human COX-2 mRNA expression in rectal xenograft models.

Tissue	Relative expression ratio of human COX-2^a^
Rectal xenograft	3.63 (2.46–4.23)
Lymph node	3.32 (1.51–106)
Lung	1.6 (0–56.8)

a Human COX-2 mRNA expression was quantified by reverse transcription TaqMan PCR and expressed as a relative expression ratio compared to that in *in vitro* cultured HT29 cells. Data are described as median (range).

**Table II:** Effect of celecoxib on rectal xenograft growth and lymph node and hematological metastases.

	Inhibition rate (p-value)		
	
		Metastatic organ	
		
Celecoxib (ppm)	Rectal xenograft	Lymph node	Lung
150	33.0 (0.00022)	86.7 (0.0263)	− (0.263)
750	46.4 (0.0000292)	90.3 (0.00638)	53.3 (0.0107)
1500	64.3 (0.000109)	96.0 (0.000894)	78.3 (0.00022)

Inhibition rate in rectal xenograft was calculated from the median weight of xenograft derived from vehicle and celecoxib-treated mice. Metastasis inhibition rate was calculated from the median number of metastatic HT-29 cells in the organ bearing rectal xenograft treated with vehicle and celecoxib. The number of tumor cells was assessed by amplification of human β-globin gene by TaqMan PCR.

**Table III:** Effect of celecoxib on PGE_2_ production, angiogenesis and apoptosis induction in human colon cancer xenograft.

	Median (range)			
			
	Vehicle (n=10)	1500 ppm celecoxib (n=10)	P-value	Relative value^a^
PGE_2_ production (ng/ml)	1647 (1125–13030)	100.3 (8.14–509)	0.000157	0.684
MVD counting (/mm^2^)	56 (32–80)	27 (9–42)	1.33×10^−12^	0.482
Apoptosis index (%)	1.64 (0.82–8.2)	4.1 (1.64–11.5)	3.00×10^−14^	2.5

a Relative value is calculated as the value in celecoxib-treated mice compared to that in vehicle-treated mice.
